# Molecular Cloning and 3D Structure Modeling of APEX1, DNA Base Excision Repair Enzyme from the Camel, *Camelus dromedarius*

**DOI:** 10.3390/ijms13078578

**Published:** 2012-07-10

**Authors:** Farid Shokry Ataya, Dalia Fouad, Ajamaluddin Malik, Hesham Mahmoud Saeed

**Affiliations:** 1 Department of Biochemistry, College of Science, King Saud University, P.O. Box 2455, Riyadh 11451, Saudi Arabia; 2 Department of Molecular Biology, Genetic Engineering Division, National Research Center, Dokki, Cairo 12311, Egypt; 3 Department of Zoology, College of Science, King Saud University, P.O. Box 22452, Riyadh 11459, Saudi Arabia; E-Mail: dibrahim@ksu.edu.sa; 4 Protein Research Chair Lab, Department of Biochemistry, College of Science, King Saud University, P.O. Box 2455, Riyadh 11451, Saudi Arabia; E-Mail: amalik@ksu.edu.sa; 5 Genome Research Chair Lab, Department of Biochemistry, College of Science, King Saud University, P.O. Box 2455, Riyadh 11451, Saudi Arabia; E-Mail: hsaeed1@ksu.edu.sa; 6 Department of Biotechnology, Institute of Graduate Studies and Research, Alexandria University, P.O. Box 832, Alexandria 21526, Egypt

**Keywords:** Ape1/Ref-1/APEX1, 3D structure modeling, DNA repair, BER, cloning, molecular characterization, qPCR, one-humped camel

## Abstract

The domesticated one-humped camel, *Camelus dromedarius*, is one of the most important animals in the Arabian Desert. It is exposed most of its life to both intrinsic and extrinsic genotoxic factors that are known to cause gross DNA alterations in many organisms. Ionic radiation and sunlight are known producers of Reactive Oxygen Species (ROS), one of the causes for DNA lesions. The damaged DNA is repaired by many enzymes, among of them Base Excision Repair enzymes, producing the highly mutagenic apurinic/apyrimidinicsites (AP sites). Therefore, recognition of AP sites is fundamental to cell/organism survival. In the present work, the full coding sequence of a putative *cAPEX1* gene was amplified for the first time from *C. dromedarius* by RT-PCR and cloned (NCBI accession number are HM209828 and ADJ96599 for nucleotides and amino acids, respectively). cDNA sequencing was deduced to be 1041 nucleotides, of which 954 nucleotides encode a protein of 318 amino acids, similar to the coding region of the APEX1 gene and the protein from many other species. The calculated molecular weight and isoelectric point of cAPEX1 using Bioinformatics tools was 35.5 kDa and 8.11, respectively. The relative expressions of *cAPEX1* in camel kidney, spleen, lung and testis were examined using qPCR and compared with that of the liver using a 18S ribosomal subunit as endogenous control. The highest level of *cAPEX1* transcript was found in the testis; 325% higher than the liver, followed by spleen (87%), kidney (20%) and lung (5%), respectively. The cAPEX1 is 94%–97% similar to their mammalian counterparts. Phylogenetic analysis revealed that cAPEX1 is grouped together with that of *S. scrofa*. The predicted 3D structure of cAPEX1 has similar folds and topology with the human (hAPEX1). The root-mean-square deviation (rmsd) between cAPEX1 and hAPEX1 was 0.582 and the Q-score was 0.939.

## 1. Introduction

Living organisms are continuously exposed to intrinsic and extrinsic agents that, if not treated properly, may result in mutation and cell death. Many enzymes are committed in the clearance of such compounds; among them are Phase I and Phase II drug metabolizing enzymes. The clearance of xenobiotics involves both activation (Phase I) and detoxification (phase II) reactions. One of the most life-threatening compounds is a group of compounds collectively known as reactive oxygen species; ROS (such as hydroxyl radical, superoxide anion and hydrogen peroxide), which is formed during the activation step. ROS is also formed by exposure to natural sunlight and from some metabolic reactions. It targets DNA, causing the generation of oxidized bases with high frequency and gross DNA alterations, thereby initiating carcinogenesis [1–3]. Under normal physiological conditions, the DNA of each mammalian cell is damaged between 10^4^ and 10^5^ times daily, and this number can be increased substantially by stresses [4–7]. Six DNA repair pathways have been reviewed by Damia and D’Incalci (2007) [8]; the most interesting for us is the Base Excision Repair (BER), because it is responsible for repairing most damage to bases in DNA. Many enzymes are involved in the BER pathway; among them DNA glycosylases which act sequentially to repair damage generating on an abasic site that can be converted to a single-stranded break by specific endonuclease [9].

APEX1/Ape1/Ref-1/APX or HAP1(EC 4.2.99.18) is an abundant, multifunctional and relatively stable mammalian BER enzyme that plays a crucial role in the regulation of the cellular response to oxidative stress [10], and controls cellular proliferative rates and maintains genome stability [11–14]. APEX1 contains a nuclear localization sequence as well as a nuclear export sequence [10]. So, it is mainly localized in the nucleus and may be translocated to the mitochondria, cytoplasm and endoplasmic reticulum after *S*-nitrosation by nitric oxide [15–17]. It catalyses many enzymatic reactions; the most studied is the endonuclease and acts as a redox co-activator [10]. APEX1 functions as an apurinic/apyrimidinic (AP) endodeoxy ribonuclease in the BER pathway of DNA lesions induced by oxidative and alkylating agents. It interacts with several proteins in both BER pathways, including 8-oxoguanine DNA glycosylase (OGG1), X-ray cross-complementing-1 (XRCC1), proliferating cell nuclear antigen (PCNA), Flap endonuclease 1 (FEN1), and polymerase b [10,18]. Following removal of the damaged base by a DNA glycosylase, APEX-1 initiates DNA repair by catalyzing hydrolytic incision of the phosphodiester backbone immediately adjacent to the AP site, generating a normal 3′-hydroxyl group and an abasic deoxyribose-5-phosphate. The deoxyribose 5′-phosphate moiety is removed by subsequent enzymes of the BER pathway which include a deoxyribose-phosphodiesterase; a DNA polymerase replaces the missing nucleotide and a DNA ligase joins the phosphodiester backbone [10,19].

APEX-1 does not act only on the AP sites in the dsDNA, but also on lesions in DNA/RNA hybrids, R-loop structure of ssDNA, and RNA molecules. It also has a 3′–5′ exoribonuclease activity on mismatched deoxyribonucleotides at the 3′ termini of nicked or gapped DNA molecules during short-patch BER and possesses a DNA 3′ phosphodiesterase activity for removing possible 3′ side-bound blocking agents (such as phosphoglycolate) of DNA strand breaks [20]. It also has a reversible nuclear redox activity; hence the name redox factor; Ref, controls the intracellular redox state by inhibiting ROS production, acts as a redox coactivator for the DNA binding of a number of transcription factors such as AP-1, c-Fos and c-Jun [21,22], NF-κB [23], p53 [10], Egr-1 [24], and as a transcriptional repressor for human parathyroid hormone and renin genes [25]. A proposed fourth function of APEX1 on RNA metabolism and gene expression has been recently discussed [26,27].

The structure of APEX1 involves two functionally distinct catalytically active regions (*i.e.*, both function independently in their actions); the *N*-terminal and the *C*-terminal regions [28]. The *N*-terminal region (residues 1–127) contains a nuclear localization signal that direct the protein to the nucleus, and five lysine residues that play a critical role in the interaction between APEX1 and RNA and in protein-protein interaction [29]. On the other hand, the *C*-terminal region (residues 61–318) exerts the AP-endonuclease enzymatic activity [10].

Impaired APEX-1 activity results in unrepaired AP sites that lead to DNA strand breaks, apoptosis, and an increase in cytotoxicity [30]. So, the combined DNA repair activity of different glycosylases and APEX serves to protect the cell from death produced by the cytotoxic and mutagenic AP sites [31].

The domesticated Arabian camel represents the main source of meat in Arabian Desert, besides its high cultural and economical values. The Arabian Desert, especially the Middle East Gulf Region, is characterized by its hot weather and strong sunlight. Clinical data indicates that although the camel is exposed for most of its life to such conditions and to other natural carcinogens, it is scarcely develops tumors (unpublished data from authorized veterinarians). Although several studies have been undertaken on DNA repair in prokaryotic and eukaryotic organisms, no researches have been done on the camel. The aim of this work was to identify the sequence of *APEX1* gene, its amino acid sequence and modeled 3D structure similarity with the human homologue and define the tissue of the highest APEX1 expression. Such work is the first step in a series of research works in the camel that could end in identifying the genes of DNA repair, ROS elimination and Phase II detoxification in the camel [32–34] and may lead to understand how the camel, as an example of naturally living mammals in the desert, can live in such harsh conditions.

## 2. Results

### 2.1. Cloning and Characterization of Full Coding Region of *cAPEX1* cDNA from *C. dromedarius* Liver

**Figure 1:**
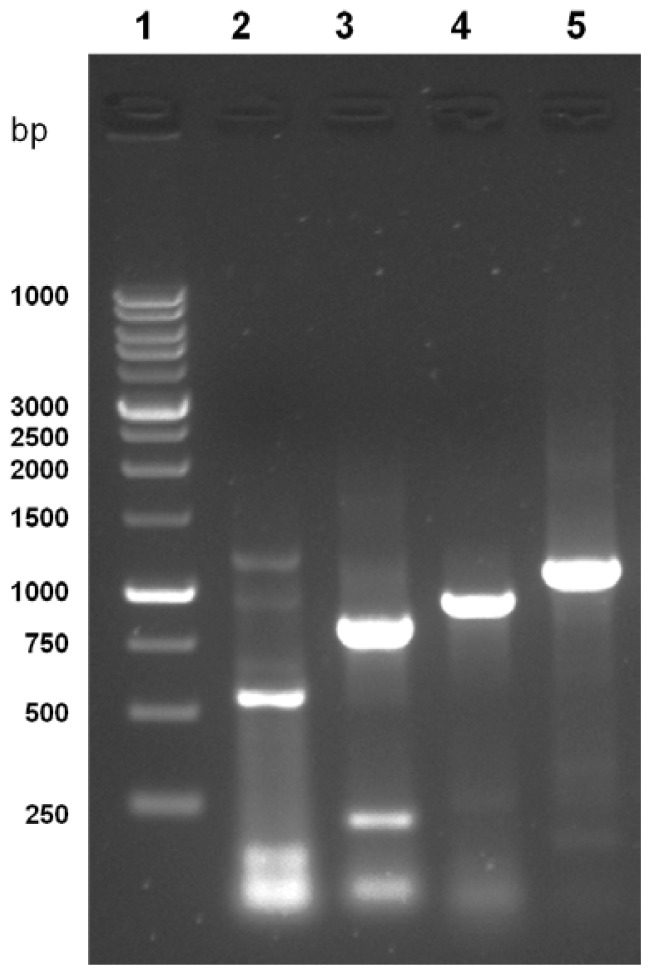
Agarose gel electrophoresis (1.2%) of the PCR products of *C. dromedarius APEX1*. Lane 1 contains 1 kb DNA molecular weight, lanes 2 to 5 the PCR products of APF1/APR1, APF2/APR2, APF2/APR3 and APF1/APR3, respectively.

**Figure 2:**
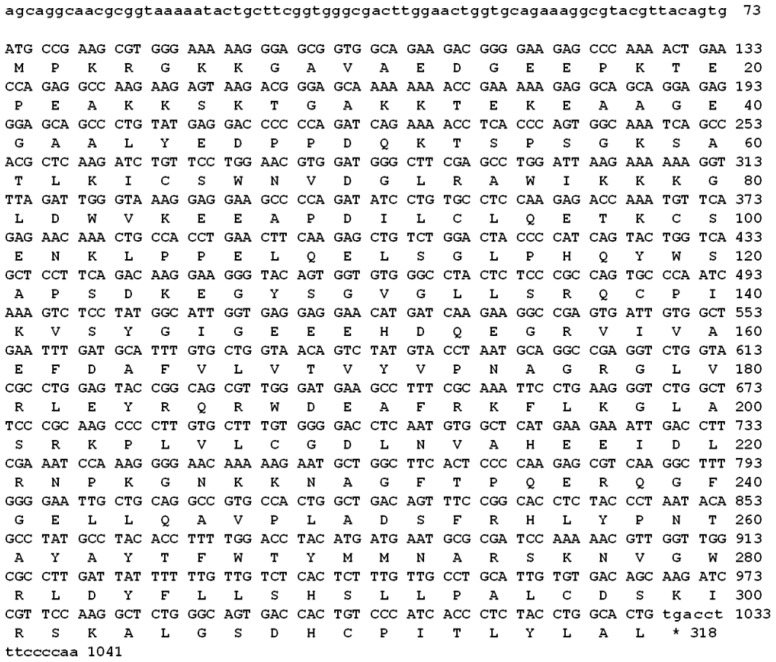
The nucleotide sequence and the deduced amino acids of the cloned *cAPEX1*. The sequences were submitted to NCBI GenBank (accession number HM209828 and ADJ96599, respectively).

A PCR-based technique was used in order to isolate the full length of *cAPEX1*. Specific primers were designed from the most conserved region of the available sequencing data in GenBank. Four cDNA fragments were amplified by RT-PCR using different primer couples (listed in the Experimental Section 4.2). The amplified cDNA fragments were electrophoretically separated on 1.2% agarose gel and their sizes were compared with the standard molecular weight ladder (Figure 1). These fragments were cut from the gel, ligated in pGEM-T Easy plasmid vector and cloned in *E. coli*. The white colonies were selected and the presence of the insert in the plasmid was confirmed by colony PCR, then the plasmids having each insert were purified from liquid medium. The inserts were sequenced using T7 and SP6 primers. The sequences of all fragments were matched and aligned by the Seqman Program [35]. The complete sequence, consisting of 1041 bp (Figure 2), represents the first cloned camel’s *APEX1*. It covers the full coding region of 954 bp and, compared with corresponding regions from different organisms, is preceded by 5′ untranslated region. Our sequence was submitted in the gene bank with the accession number HM209828. The nucleotide BLAST analysis for the coding region of *cAPEX1* showed that it shared high similarity (94%–86%) with *cAPEX1* from other mammals: horse *E. caballus* (94%), pig *S. scrofa* (94%), cattle *B. taurus* (92%), human *H. sapiens* (91%), chimpanzee *P. troglodytes* (91%), rhesus monkey *M. mulatta* (90%), Sumatran orangutan *P. abelii* (90%), guinea pig *C. porcellus* (89%), dog *C. familiaris* (87%) and house mouse *M. musculus* (86%).

### 2.2. Amino Acid Composition of cAPEX1

The deduced amino acid sequence was found to form an open reading frame of 318 amino acid residues (Figure 2). The amino acid sequence was submitted in the gene bank with the accession number ADJ96599. The molecular analysis of the 318-amino acid sequence of cAPEX1 using the PROTEAN program [36] showed that this protein has a molecular weight of 35.5 KDa, pI 8.11 and molar extinction coefficient 56030% ± 5%. The predicted protein contains 114 charged amino acid (35.85%), 103 hydrophobic (32.39%), 43 acidic (13.52%), 46 basic (14.47%) and 71 polar amino acids (22.33%). The complete amino acid analysis and chemical composition of the predicted protein are illustrated in Table 1.

### 2.3. Multiple Sequence Alignment and Phylogenetic Analysis

**Figure 3:**
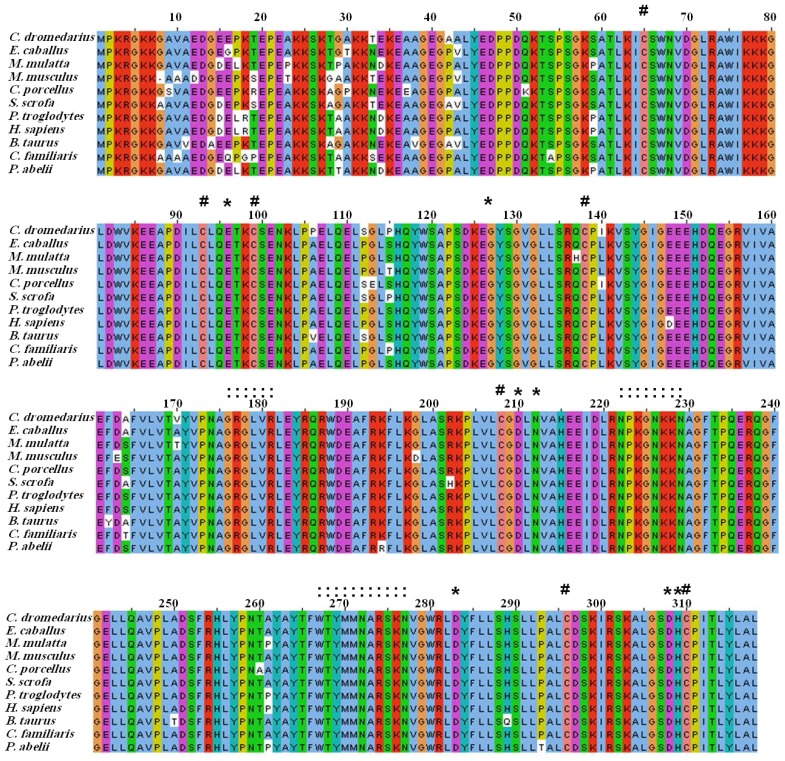
Amino acid sequence alignment of Camel APEX1 and potentially related proteins from the GenBankTM data base. The alignment was generated with the MAFFT Multiple Sequence Alignment program. The *N*-terminal critical lysine residues (K24, K25, K27, K31 and K32) are present in the camel. # conserved cysteine; * catalytic and enzymatically important residues;: residues involved in DNA backbone interactions.

**Figure 4:**
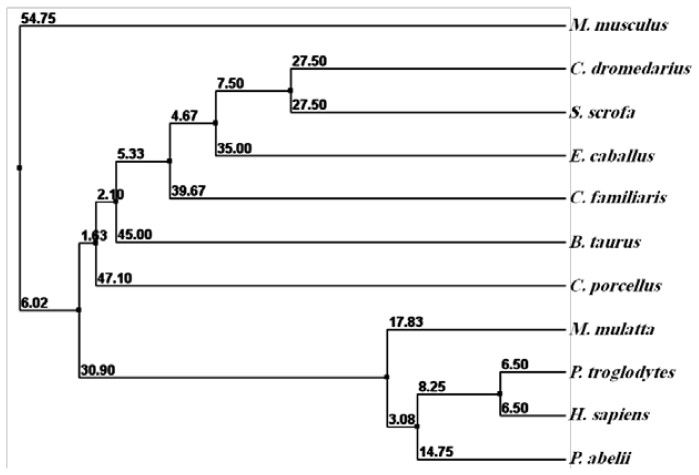
The phylogenetic tree of cAPEX1 and potentially related genes. The protein sequence of APEX1 was compared with other mammalian sequences of the GenBankTM database. The alignment was generated with the BLOSUM62 from MAFFT Multiple Sequence Alignment.

The comparison between the predicted amino acid sequence of cAPEX1 and the sequences from the most similar APEX1 from different organisms was carried out. The amino acid sequence of cAPEX1 was aligned with 10 different mammalian APEX1 by ClustalW [37,38] (Figure 3, Table 2). The protein BLAST analysis showed higher identity (97%–94%) with *cAPEX1* from other mammals: *E. caballus* (97%), *S. scrofa* (97%), *B. taurus* (96%), *C. familiaris* (96%), *H. sapiens* (95%), *P. abelii* (95%), *P. troglodytes* (95%), *M. mulatta* (95%), *M. musculus* (95%) and *C. porcellus* (94%). The phylogenetic analysis and the high sequence identity of the examined proteins confirms the close evolutionary relationship between *S. scrofa* and *C. dromedarius* (Figure 4).

### 2.4. Secondary and 3D Structure Modeling of cAPEX1

**Figure 5:**
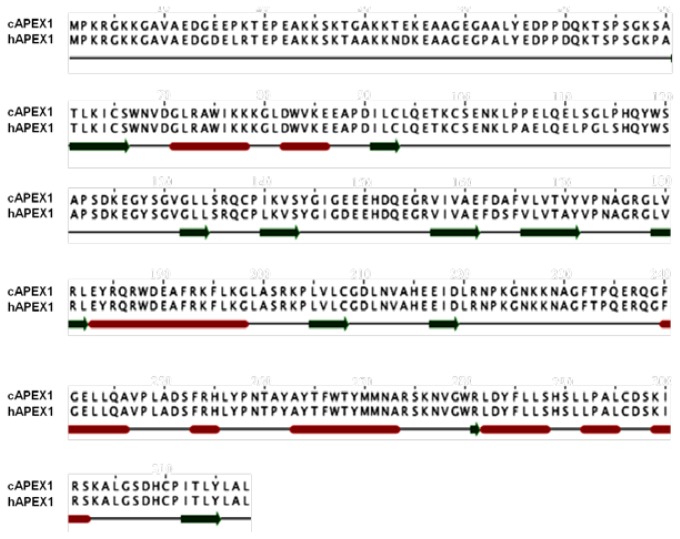
The secondary structure annotation sites of the cAPEX1 and hAPEX1 sequences using Jalview program. Red cylinders and green arrows indicated helix and β-sheet, respectively.

A prediction of the secondary structure analysis of cAPEX1 was carried out using the Jalview program [38] and compared with the human homolog, hAPEX1 (Figure 5). The predicted structure suggested that this protein is composed of 9 helices and 11 β-sheets. The secondary structure of cAPEX1 is almost the same like hAPEX1.

**Figure 6:**
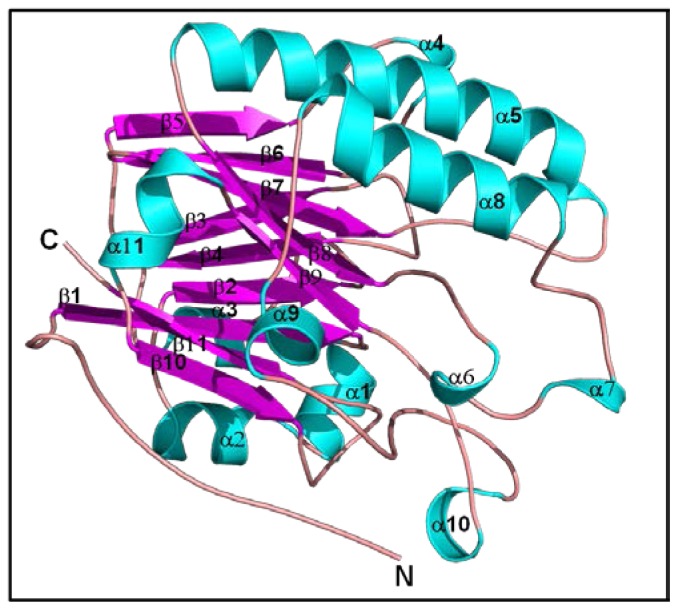
Predicted 3D structure model of cAPEX1. The 3D structure model of cAPEX1 was predicted using a Swiss-model server.

The 3D structure of cAPEX1 was modeled using homology structure modeling on a Swiss model server [39]. To predict the 3D structure of cAPEX1, 3D structure was applied at 2.15 °A of hAPEX1 (PDB ID 3U8U), which shared 95% sequence identity. The modeled 3D structure of cAPEX1 had very similar fold and topology as those of human APEX1 (Figure 6). The modeled 3D structure of cAPEX1 is globular a/b rich protein, containing 11 β-sheets at the core which is surrounded by 11 helical structure.

### 2.5. Comparison between Structure of Camel and Human APEX1

**Figure 7:**
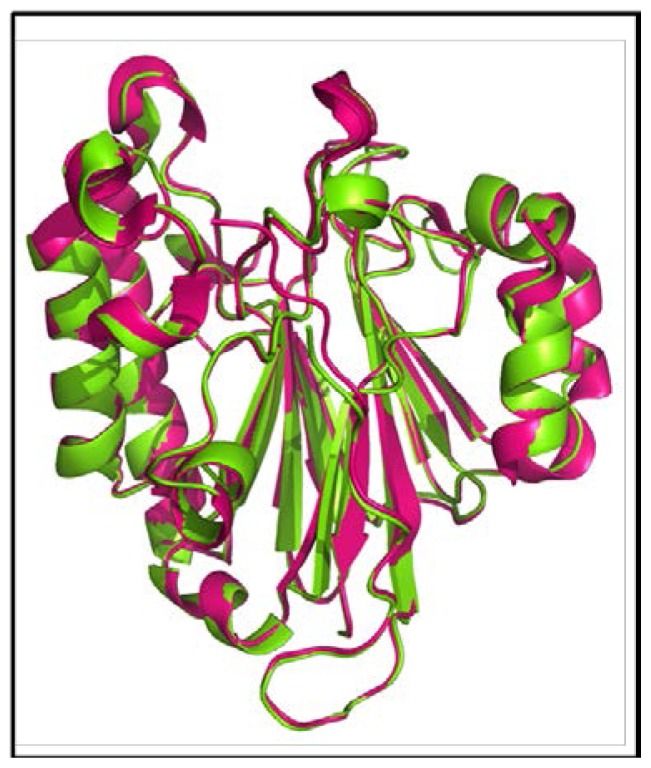
Superimposed 3D structure of camel cAPEX1 (green) with human hAPEX1 (pink). The superimposition indicated very high similarity between the structures of cAPEX1 and hAPEX1.

**Figure 8:**
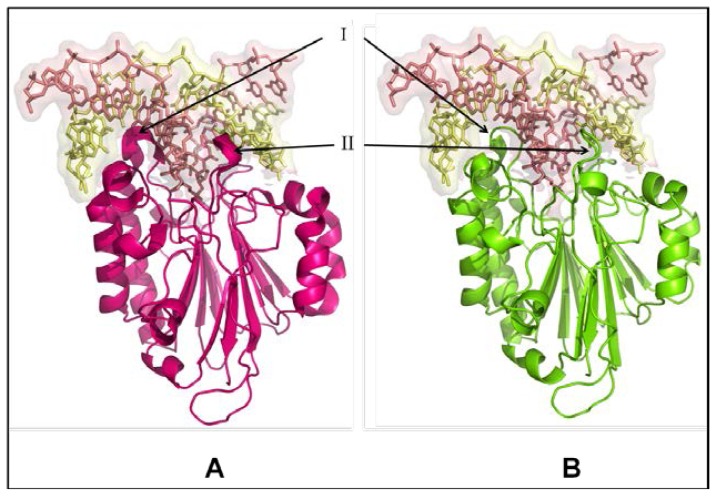
DNA binding site in human (**A**) and camel (**B**) APEX1. The upper part of the figure (shaded in light pink) represents the DNA and lower part is the 3D structure APEX1. The surface exposed helices around DNA binding sites are slightly different (I and II).

**Figure 9:**
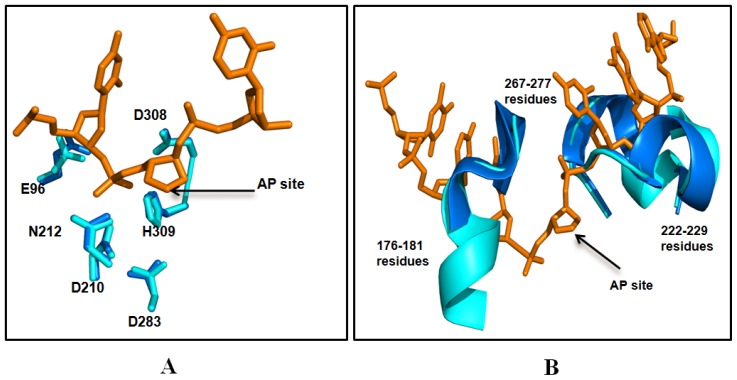
Comparison of the catalytic and enzymatically important residues (**A**) in camel (marine blue) and human (cyan color) APEX1 and DNA structure (orange color), and the APEX-DNA backbone interaction residues (**B**) in camel and human APEX1. The active sites of the cAPEX1 from the two organisms contain the highly conserved E96, N210, N212, D283, D308 and H309 residues. The residues are numbered according to the amino acid sequence of *C. dromedarius* (accession number ADJ96599). This comparison indicated very high identity between the predicted active site of cAPEX1 and that of hAPEX1.

The structural similarity of camel APEX1 with human APEX1 was studied by superimposing their structures using the Pymol program (http://pymol.sourceforge.net) [40]. The folds and topology of modeled cAPEX1 is very similar to hAPEX1 (Figures 7 and 8a,b). The quality of the predicted structure of cAPEX1 was compared with hAPEX1 as a template (PDB id 1DEW), using PDBeFold on an EMBL-EBI server [41]. 275 residues of modeled 3D structure on cAPEX1 were aligned with 3D structure of hAPEX1, with an overall rmsd of 0.582. Major sequence and structural differences were found in the helical loop regions. The surface exposed helices around the DNA binding sites are slightly different (Figure 8a,b). Residues involved in the catalysis and interaction with the backbone of DNA superimposed very well in cAPEX1 and hAPEX1 (Figure 9a,b). The Q-score is an important parameter to assess the similarity of the homologous structures which represents the quality of structure recognition and superimposition. Identical structures have a Q-score of 1. The Q-score of cAPEX1 and hAPEX1 superimposition was 0.939, indicating high structural identity. The P- and Z-scores of the 3D structure of cAPEX and hAPEX were 48.5 and 22.6, respectively. Therefore, Q-, P- and Z-scores indicate that the modeled structure of cAPEX1 is similar to hAPEX1.

### 2.6. Expression of cAPEX1 Gene by Real Time PCR

**Figure 10:**
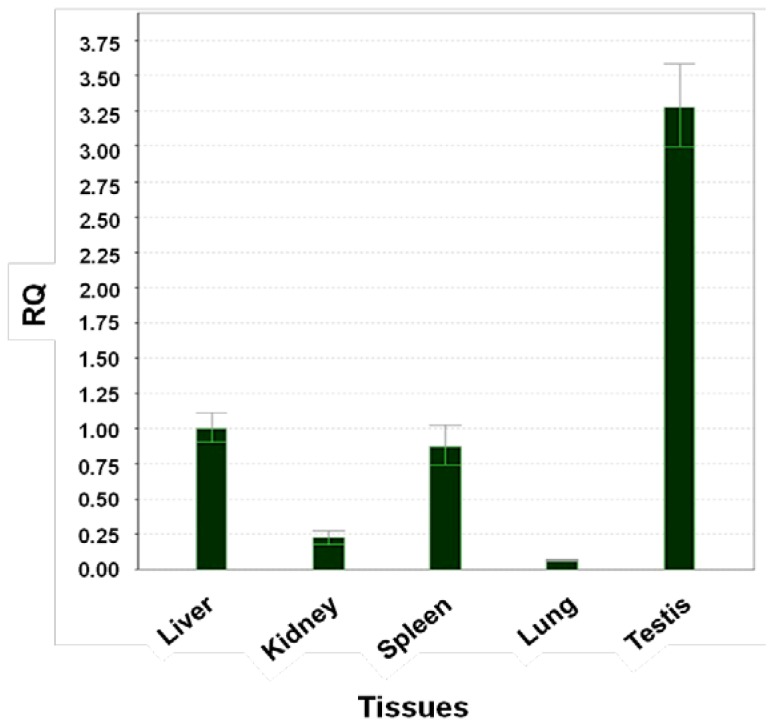
Expression of cAPEX1 using Real time PCR and cDNA from different camel tissues. The results are expressed relative to liver as calibrator and using the 18S ribosomal subunit as housekeeping gene.

The level of expression of APEX1 in five camel tissues is examined using Real Time-PCR. The primers were designed to amplify 194 base pairs and the experiment conditions were adjusted to the best annealing between primers and cDNA, to eliminate the primer dimer, self dimer or hairpin form and to amplify only one band representing part of *cAPEX1*. The expression of APEX1 in the liver was taken as a reference sample (calibrator) and the expression of 18S ribosomal subunit as a housekeeping gene (endogenous control). The relative expressions of APEX1 in the kidney, spleen, lung and testis were compared with that of the liver. The expression level in testis was 3.25 fold (325%) higher than in the liver, followed by spleen (87%), kidney (20%) and lung (5%), relative to liver (100%), respectively (Figure 10).

## 3. Discussion

In the past decade, attention on biochemical aspects of camel research has been focused. The camel is a culturally and economically important animal, especially in the Middle East and the north African region, where the camel represents the main source of meat and its milk is used as traditional medicine for the maintenance of health and in the treatment of various diseases. Many studies have been done on the camel, most of them focused on the composition of its meat and milk, as well as on the processes of its adaptation for the harsh environment of the desert. The biochemical pathways of the camel are the least studied pathways in mammals. Nevertheless, the few serious studies that have examined the camel revealed surprising results. Recent publications suggested that camel milk has antidiabetic [42,43], antischistosomal [44], anti-HCV [45], and/or apoptotic activity [46].

Camels have special physiological and anatomical characteristics, enabling them to live in the very hot and dry climate under direct exposure to burning sunlight and natural UV radiations. Generally, ionic radiation causes damage and/or alterations in the DNA of the living cells, resulting in mutation, cancer and cell death. To the best of the author’s knowledge, no studies have been performed on the camel to examine how the camel can overcome the deleterious effect produced by direct sun exposure and how it can repair probable DNA lesions and other lesions induced by different DNA-damaging agents. This brings us to initiate the cloning and characterization of the camel’s multifunctional DNA-repair enzyme, cAPEX1, and to predict its 3D structure modeling, and compare it with the human homolog. The DNA repair mechanism relays on the concomitant action of many enzymes. The study of a single DNA repair enzyme cannot confirm the relationship between the low tendency of evolving tumors in the camel and the expression of cAPEX1. This work, together with our ongoing work on camel DNA repair enzymes (OGG1, Neil, MPG), may lead to an understanding of the similarities between camel and human DNA repair enzymes which could help, using the camel as a model for mammals living in the desert to elucidate the mechanism of adaptation against high ionic radiation, temperature and dryness.

In this study, cAPEX1 from the one-humped camel was cloned for the first time. Our results showed amplification of a cDNA fragment of 1041 bp covering the whole coding region using a primer set spanning the gene (Figure 2). This sequence contains part of the 5′ untranslated region, the start and the stop codons. The open reading frame is composed of 954 bp which is comparable with the sequences from most mammalian species (Figure 3) and codes a deduced protein of 318 amino acid residues of 35.5 kDa. Our *cAPEX1* sequence has been matched with several APEX1 sequences in GenBank and submitted in the genbank/NCBI database with the accession number HM209828 and ADJ96599 for nucleotides and protein, respectively.

The comparison between the predicted amino acid sequence of cAPEX1 and the sequences of conserved domains from different organisms indicated that this protein belongs to superfamily [cd09087]. It is a large family of proteins, including Mg^2+^ dependent endonucleases and a large number of phosphatases involved in intracellular signaling (NCBI database). Divalent cation binding residues (Mg^2+^ and Mn^2+^) were found conserved in cAPEX1 at 68, 96, 210, 212, 308 and 309.

The amino acid alignment of the cAPEX1 and 10 mammalian species has shown that the *C*-terminus is more conserved than the *N*-terminus. Despite of this finding, it has been reported that the *N*-terminal domain may play a role in the fine regulation of the AP endonuclease activity of APEX1. Among the 10 highly conserved lysine residues located in site K3, K4, K6, K7, K24, K25, K27, K31, K32 and K35, only five of which (K24, K25, K27, K31 and K32), are involved in the interaction of APEX1 with both RNA and NPM1 [29].

The cAPEX1 contains the seven highly conserved cysteine residues located at 65, 93, 99, 138, 208, 296 and 310 in all the mammalian APEX1. It has been noted that cysteine at position 65 is involved in redox activity as the C65A mutant of hAPEX1 eliminates the redox activity while introduction of cysteine at corresponding position in zebrafish APEX1 leads to a gain of redox activity [47,48]. The structural analysis has shown that the first 43 amino acids of cAPEX1 are relatively non-conserved, while the rest of the protein has a globular structure. This coincides with the findings of other investigators [49,50].

From the two organisms, the AP sites of the cAPEX1 and its surrounding contain the highly conserved E96, N210, N212, D283, D308 and H309 residues. This comparison indicated very high identity between the predicted active site of cAPEX1 and that of hAPEX1.

This result coincides with the sequence of APEX1 from different mammals. Its crystallization data indicates that this region is highly flexible and does not form alpha or beta sheets. Nuclear localization signal sequence (GAVAED) is located at the *N*-terminus at residues 8–13. Nuclear export signal (ICSWNVDGLRAWIKKKG) is located on amino acid 64–80 and controlled by nitrosylation [10,16]. Moreover, the mitochondrial targeting sequence (HSLLPALCDSKIRSKALGSDHCPITLYLAL) is also conserved on C-terminus at the amino acids 289–318.

Proteins with similar amino acid sequences have a tendency to adopt similar 3D structures. Therefore, it is possible to predict the 3D structure of the putative *C. dromedarius* APEX1, using the known published *H. sapiens* APEX1 crystal structure as a template for modeling our predicted enzyme [49,50].

APEX1 is a 35 KDa monomeric protein, consisting of two symmetrically-related domains with similar topology. The predicted cAPEX1 was found very similar to hAPEX1. The DNA binding site is located at the top of a/b sandwich and the predicted catalytic site is surrounded by a helical-loop region (Figure 8a,b). Based on the high degree of sequence conservancy, the same catalytic residues, and the metal binding residues in both cAPEX1 and hAPEX1, it likely that cAPEX1 is functionally very similar to hAPEX1. To adapt in high temperature and dryness, cAPEX1 may be more thermodynamically stabilized during the course of evolution through the synthesis of some stabilizing agents or heat shock proteins.

Our findings suggest that APEX1 is highly expressed in testis followed by liver, as indicated by qPCR. This high expression level is expected as APEX1 and other DNA repair machinery is important to correct mistakes and oxidized bases in DNA of the highly dividing cells, like in testis. Also it is expected to be found in the liver where most of the metabolic processes are performed with the possibility of ROS production.

## 4. Experimental Section

### 4.1. Samples and Materials

Fresh camel tissues (liver, kidney, spleen, lung and testis) from adult males were obtained immediately after slaughtering from Riyadh Main Slaughterhouse. The camel tissues were instantly submerged in RNAlater solution (Qiagen, Ambion, Courtabeuf, France) to avoid RNA degradation (Qiagen, France) and stored at −80 °C. Cultivation of *Escherichia coli* strains was done in Luria-Bertani (LB) medium supplemented with 100 μg/mL ampicillin, unless otherwise mentioned.

### 4.2. Oligonucleotide Design

Highly conserved regions of APEX1 genes from GenBank database; mostly from *S. scrofa* and *E. caballus*, were selected to design series of oligonucleotide primers (Table 3). Combinations between primer couples were tested at different temperatures to give specific bands to cover the full coding region. Two primers were also designed for the qPCR, namely AP1qF and AP1qR, to amplify a product of 194 bp. The amplification product length and the optimum annealing temperature of each primer couples are listed in Table 3.

### 4.3. RNA Extraction, cDNA Synthesis and Reverse Transcription PCR

RNAlater treated liver, kidney, spleen, lung and testis were used to prepare the total RNA. Approximately 50 mg of each tissue was homogenized in RTL lysis buffer (Qiagen, France) supplemented with 1% 2-mercaptoethanol according to manufacturer’s instruction using a rotor-stator homogenizer (Medico Tools, Switzerland). Total RNA of the homogenized tissue was extracted using AllPrep DNA/RNA Mini kit (Qiagen, France, Cat # 80204). Elution was performed with 50 μL nuclease-free water. RNA was quantified by NanoDrop-8000 (Thermo, DE, USA) and RNA integrity was assessed on denaturing formaldehyde agarose gel (1%) electrophoresis. Two microgram of total RNAs were subjected to reverse transcription to single-stranded cDNA using ImProm-II Reverse Transcription System (Promega, Cat # A3800, USA) with the following cycling conditions: 96 °C for 1 min, followed by 40 cycles at 94 °C for 30 s, 65 °C for 30 s, and 72 °C for 1 min.

### 4.4. Polymerase Chain Reaction and Cloning

PCR (50 μL volume) was done in gradient manner using annealing temperatures ranging from 50–60 °C. In the reaction mixture, 25 μL of GoTaq^®^ Green Master Mix (Promega, Cat # M712c), 5 μL of cDNA, 3 μL of each forward and reverse primers (30 pmol) and 14 μL nuclease free water was added. The amplification condition was set as follows: one cycle at 95 °C for 45 s followed by 40 cycles at 94°C for 30 s, 50–60 °C for 45 s and 68 °C for 90 s. Final extension was carried out at 72 °C for 5 min to make blunt end product. Qualitative and quantitative analysis of PCR products were done on 1.0%–1.5% agarose gel electrophoresis.

Ethidium bromide stained PCR product of the estimated size on agarose gel was excised. The PCR product was extracted from the gel using QiAquick gel extraction kit (Qiagen, Cat # 28706). The PCR product was estimated using NanoDrop-8000 and ligated on the pGEM-T Easy vector (Promega, Cat # A1360). In order to ligate purified PCR product onto pGEM-T vector, 2 μL of purified PCR products were mixed with 1 μL pGEM-T-Easy vector (50 ng), 5 μL of 2× rapid ligation buffer and 3 units of T4 DNA ligase. The final volume of the ligation mixture was adjusted to 10 μL by nuclease-free water and the mixture was incubated at 15 °C for 16 h. Transformation of *E. coli* JM109 competent cells was carried out according to Sambrook *et al*. [51]. *E. coli* harboring the recombinant plasmid were screened using selective LB agar containing isopropyl-β-d-thio-galactoside (IPTG), 5-bromo-4-chloro-indolyl-β-d-galactopyranoside (X-gal), and ampicillin. Moreover, colony PCR was conducted to screen for recombinant bacteria using T7/SP6 primers.

### 4.5. Studying Gene Expression by qPCR

The expression patterns of *cAPEX1* mRNA in 5 different tissues (liver, kidney, spleen, lung, and testis) were quantified using real-time PCR (Applied Biosystems 7500 Fast real-time PCR system). All the reactions were made in triplicates. The qPCR mixture included the cDNA, 5 pmole each AP1qF and AP1qF primers and 10 μL Fast-SYBR Green qPCR Master Mix (Applied Biosystems) in a final 20 μL reaction volume as recommended by the manufacturer. The parameters of qPCR was the following: initial denaturation at 95 °C for 3 min, amplification over 40 cycles of serial heating at 95 °C for 3 s and 60 °C for 30 s. The amplification specificity was verified by melting-curve analysis immediately after the amplification protocol by increasing the temperature to 95 °C for 15 s followed by 60 °C for 1 min and ramping the temperature of the reaction samples from 60 °C to 95 °C.

### 4.6. DNA Sequencing and Prediction of Amino Acid Sequence

PCR fragments cloned onto pGEM-T-Easy vector were sequenced by the chain termination method of Sanger *et al*. [52] either using 3730xl DNA analyzer (Applied Biosystems DNA Sequencing System) or MegaBACE 1000 DNA Sequencing System (GE Healthcare, NJ, USA). The conditions of the Chain Termination PCR were; one cycle at 95 °C for 30 s followed by 30 cycles at 94 °C for 30 s, 50 °C for 30 s and 60 °C for 1 min. The nucleotide sequencing was done from both directions and the sequences were analyzed using the Seqman PROGRAM [35]. The sequenced DNA fragment was translated using EditSeq of DNASTAR program [53] and the deduced cAPEX1 amino acid sequence was compared in the NCBI Protein Database using the BLASTP algorithm [54].

### 4.7. Multiple Sequence Alignment and Analysis of Phylogenetic Relationship

Camel cAPEX1 (accession number ADJ96599) amino acid sequence was used as template to identify homologous mammalian sequences in PSI-BLAST. Ten homologous sequences from different mammals were used for multiple sequence alignment by ClustalW on MAFFT Multiple Sequence Alignment and Jalview [37,38]. The output of MAFFT Multiple Sequence Alignment was color-coded according to their identity. The amino acid sequences of cAPEX1 and other ten mammalian APEX1 enzymes were subjected to construct a phylogenetic tree using BLOSUM62 program [37,38] from MAFFT Multiple Sequence Alignment. The sequence similarity among them was calculated by Blast2 sequence.

### 4.8. Secondary and Prediction of the 3D Structure of cAPEX1

The amino acid sequence of cAPEX1 (accession number ADJ96599) was subjected to predict its secondary and 3D structure. The secondary structure was predicted using Jalview program while the 3D was predicted using a Swiss-model server using homology structure modeling [38].

The similarities between modeled cAPEX1 structure and human APEX1, the catalytic and enzymatically important residues and DNA backbone interaction residues in cAPEX1 were superimposed on hAPEX1 using Pymol software (delino Scientific) [40]. The quality of the superimposed 3D structures was assessed using PD Be on EMBL-EBI server.

## 5. Conclusions

The isolated *C. dromedarius APEX1* represents the first full length DNA repair gene to be cloned and characterized so far from this unique animal. The primary and secondary structure of cAPEX1 and the predicted active site are very similar to human homologue. The predicted structure revealed the preservation of several key structural features, such as the substrate binding site, the nuclear localization signal sequence, the nuclear export signal and the mitochondrial targeting sequence. The highest expression was observed in testis and liver where most of the synthetic and metabolic processes are performed.

## Figures and Tables

**Table 1:** Predicted chemical composition of the cloned full length of cAPEX1 using Protean Program.

Amino acid	Number count	% by weight	% by frequency	Amino Acid	Number count	% by weight	% by frequency
**Ala (A)**	27	5.41	8.49	**Met (M)**	3	1.11	0.94
**Cys (C)**	7	2.03	2.20	**Asn (N)**	10	3.21	3.14
**Asp (D)**	16	5.19	5.03	**Pro (P)**	20	5.47	6.29
**Glu (E)**	27	9.82	8.49	**Gln (Q)**	10	3.61	3.14
**Phe (F)**	9	3.73	2.83	**Arg (R)**	10	3.61	3.14
**Gly (G)**	26	4.18	8.18	**Ser (S)**	20	4.91	6.29
**His (H)**	6	2.32	1.89	**Thr (T)**	12	3.42	3.77
**Ile (I)**	9	2.87	2.83	**Val (V)**	16	4.47	5.03
**Lys (K)**	30	10.83	9.43	**Trp (W)**	7	3.67	2.20
**Leu (L)**	11.16	11.01	11.16	**Tyr (Y)**	12	5.52	3.77

**Charged amino acids (RKHYCDE)**					**114**	**42.74**	**35.85**
**Acidic (DE)**					**43**	**15.01**	**13.52**
**Basic (KR)**					**46**	**17.87**	**14.47**
**Polar (NCQSTY)**					**71**	**22.70**	**22.33**
**Hydrophobic (AILFWV)**					**103**	**31.30**	**32.39**

**Table 2:** Comparison of cAPEX1 and other APEX1 enzymes from different mostly similar organisms. The comparison included a number of amino acid residues, percent identity, E-value and isoelectric point (pI).

APEX1	(NCBI Ref. Seq)	Amino acid residues	Total score	Identity (%)	Positive (%)	Gap (%)	*E*-value	pI
*Camelus dromedarius*	ADJ96599	318	656	100	100	0	0.00E+00	8.32
*Equus caballus*	XP_001505181.1	318	635	97	97	0	0.00E+00	8.51
*Sus scrofa*	NP_001132943	318	622	97	98	0	0.00E+00	8.04
*Bos taurus*	NP_788782	318	613	96	97	0	1.00E−180	8.32
*Canis lupus familiaris*	NP_001138591.1	318	610	96	97	0	1.00E−179	8.33
*Homo sapiens*	NP_001632.2	318	606	95	97	0	3.00E−178	8.33
*Pongo abelii*	XP_002824554.1	318	605	95	97	0	6.00E−178	8.33
*Pan troglodytes*	NP_001074954.1	318	607	95	97	0	1.00E−178	8.33
*Macaca mulatta*	XP_001090240.1	318	622	95	96	0	0.00E+00	8.32
*Mus musculus*	NP_033817.1	317	619	95	96	0	0.00E+00	8.04
*Cavia porcellus*	XP_003474586	318	616	94	96	0	0.00E+00	8.32

**Table 3:** List of primers used for the amplification and expression of *cAPEX1*.

Primer	Primer sequence	Primer couple	Product (bp)	Annealing temperature
APF1	AGCAGGCAACGCGGTAAAA	APR1 APR3	548 041	58 58
APR1	GCCTTCTTGATCATGTTCCTCCTC	APF1	540	58
APF2	GGATCCATGCCGAAGCGTGGGAAAA	APR2	854	58
APR2	AGTAATCAAGGCGCCAACCAACAT	APF2	854	58
APR3	TTGGGGAAAGGTCACAGT	APF1 APF2	1041 954	55 52
AP1qF	GGTAAAGGAGGAAGCCCCAGATA	AP1qR	194	56
AP1qR	TCACCAATGCCATAGGAGACTTT	AP1qF	194	56
